# Participation in Younger and Older Adults Post-stroke: Frequency, Importance, and Desirability of Engagement in Activities

**DOI:** 10.3389/fneur.2019.01108

**Published:** 2019-10-18

**Authors:** Joan Toglia, Gulce Askin, Linda M. Gerber, Abhishek Jaywant, Michael W. O'Dell

**Affiliations:** ^1^Mercy College, Dobbs Ferry, NY, United States; ^2^Rehabilitation Medicine Department, Weill Cornell Medicine, New York, NY, United States; ^3^Department of Healthcare Policy and Research, Weill Cornell Medicine, New York, NY, United States; ^4^NewYork-Presbyterian Hospital, New York, NY, United States; ^5^Department of Psychiatry, Weill Cornell Medicine, New York, NY, United States

**Keywords:** outcome assessment, social participation, stroke, rehabilitation, subjective appraisal, community participation indicators, Stroke Impact Scale

## Abstract

**Purpose:** To characterize and compare frequency and subjective dimensions of post-stroke participation in younger (<65) and older adults (>age 65), in social, productivity and leisure activities, 6 months post-inpatient rehabilitation. Secondary aims included exploration of demographic and clinical factors influencing desire for increased participation and comparison of two measures of participation.

**Methods:** A prospective cohort study of people with stroke (*n* = 99) who were identified during their inpatient rehabilitation stay and followed-up 6 months post-discharge with telephone interviews using two self-report participation measures. The Stroke Impact Participation subscale (SIS-P) measured the frequency of perceived limitations in social, leisure, productive activities and extent of stroke recovery. The Community Participation Indicators (CPI) examined activity frequency, importance, and desire for increased activity engagement. Descriptive statistics were used to summarize demographic variables and characterize SIS-P and CPI items. Differences between age groups on individual items were examined. Associations between measures and demographic variables were explored.

**Results:** Both groups reported a wide variation in participation restrictions that was not associated with stroke severity and weakly associated with discharge functional status (rho = 0.20–0.35). There were no significant differences between age groups in CPI frequency (for 18/19 items), or the SIS-P. However, there was a trend toward more participation restrictions on the SIS-P among those <65 (*p* = 0.07). Younger adults (*n* = 46; median age = 53) were significantly more likely to indicate that they were not doing selected activities enough on the CPI, compared with older adults (*n* = 56; median age = 76). While age and ethnicity were independently associated with some activities, it was not associated with other activities. The CPI and SIS-P were moderately related at a correlation of rho = 0.54, *p* < 0.001.

**Conclusion:** The CPI demonstrated value and utility in examining subjective perspectives of activity importance and desire for change for people who are 6 months post-stroke. Although the CPI and SIS-P are moderately related, subjective appraisal of participation in selected individual activities (CPI) better distinguished between age groups and provided unique and distinct information from the SIS-P.

## Introduction

Stroke is a leading cause of long-term disability. Annually, ~795,000 people experience a stroke each year in the United States (US) (1). Despite declining incidence and mortality rates, the number of people living with a disability as a result of ischemic stroke has increased by 55% between 1990 and 2016 in the US (2). Improved medical management, higher survival rates, and longer lifespans have resulted in people living longer after a stroke.

Although decreased incidence of stroke in the general population has been reported, a trend toward rising stroke occurrences among younger age groups over the past decade has been reported globally in numerous studies (3–5). For example, the number of Americans hospitalized for a stroke below age 65 increased by 49% in the last decade (6). The underlying cause of stroke incidence in younger age groups is multifactorial but increases in risk factors such as cocaine use and cannabis use appear to be associated with this trend (7).

In general, both younger and older adults report significant long-term participation restrictions following a stroke. *Participation* is widely regarded as the ultimate goal of rehabilitation following a stroke. The International Classification of Functioning, Disability and Health defines participation broadly as involvement in life situations, including roles and activities (8). The aim of rehabilitation is to help people return to their lives and fulfill roles and engage in meaningful life activities. There is little information however, comparing activity engagement in younger and older adults post-stroke, particularly among those who have completed a course of short term intensive inpatient rehabilitation (defined by a minimum of 3 h of therapy per day).

The impact of stroke disability on the lives of people who are 65 years and younger may be very different than those who are older. For example, stroke during working age years has a large socioeconomic impact due to loss of productivity, and results in people living with a long-term disability over a longer time (3, 9). Further, the relative value and meaning placed on various functional activities and social roles likely differs by age (9). Overall, the rise in the number of people living with a chronic stroke-related disability indicates a pressing need to fully understand the unique participation restrictions experienced by both younger and older people who are living with the effects of stroke.

The measurement of a complex construct such as participation presents challenges because there is a lack of consensus regarding its conceptualization and operationalization (10). It is not always clear if different participation assessments will yield consistent results. Most participation instruments examine self-reported frequency such as the amount of time one experiences limitations, or spends in social, leisure, productive activities and roles. This has been described as the objective aspect of participation, because it can be observed, easily quantified or reported, and ratings can be compared across different people or groups (10). In addition to frequency, another dimension of participation involves the subjective aspect or the person's experiences, feelings and self-perceptions. The subjective aspect of participation includes autonomy, importance of activities to the person, satisfaction, and desire for changes in participation (10, 11). Activities that are important or meaningful to a person or that the person would like to do more often, are individualized and depend on the person's preferences, interests, life roles or the context of that person's life (12). Although there are a few exceptions, the majority of participation instruments only focus on the objective (i.e., frequency) dimension of participation (10).

Comparisons of both subjective and objective dimensions of participation in people with stroke are limited in the literature. Studies that have included measures of subjective participation have focused on perceived satisfaction (11), however desire for increased participation in valued activities has not been explored. Additionally, the subjective and objective aspects of participation have not been compared in younger and older adults.

Therefore, this study aims to describe and compare self-reported participation restrictions including frequency, importance and desire for change in younger and older stroke survivors above or at and below age 65. Specifically, our aims are to (1) Compare the frequency of participation and perceived stroke recovery between age groups, (2) Describe and compare the subjective aspects of participation (activity importance, desire for change) between age groups, (3) Determine the association of participation domains with demographic and clinical variables, (4) Assess the relationship between objective and subjective aspects of participation as measured by the Stroke Impact Scale- Participation subscale (SIS-P) and the Community Participation Indicators (CPI), respectively.

## Materials and Methods

### Participants

We prospectively identified participants with a diagnosis of stroke, who were consecutively admitted to an inpatient rehabilitation unit (IRU) within a large academic medical center between July 25, 2012 and July 6, 2016. All included participants provided written informed consent to have demographic and routine clinical information obtained during their inpatient stay, entered into a stroke rehabilitation database. At the same time, they also provided consent to be contacted for a follow-up telephone interview 6 months after discharge. The study was approved by the facility's Institutional Review Board for Human Subjects Research. Inclusion criteria were the same as those for admission to the IRU and included individuals who were 18 years of age or older, who were medically able to participate in inpatient rehabilitation therapies for 3 h daily, and who had a reasonable chance of making functional gains. Those who fully completed targeted follow-up telephone measures with no more than 4% of responses missing, between 6 and 7 months following discharge from inpatient rehabilitation (*n* = 99), and were living in the community were included in this analysis.

### Participation Assessment Measures

#### The Community Participation Indicators (CPI), Part 1

The Community Participation Indicators (CPI) is a newer participation measure that was developed by Heinemann et al. using multiple stakeholder focus groups to explore what the concept of participation meant to people with disabilities (13). In addition to examining activity frequency, part 1 of the CPI accounts for individual preferences for activity engagement by examining importance and the person's desire to engage in each activity more often.

The CPI (part 1) includes 20 items related to productive roles activities, social activities and relationships, recreations and leisure (13). For 19 items, respondents rate each item on (1) frequency of engagement on a scale of either 1 (none) to 5 or 6 (high frequency) in terms of number of hours, days or times or times per week depending on the activity type; (2) whether it was important (yes/no), and (3) to what extent they were doing the activity too much, enough, or not enough. The ratings of “enough” and “too much” were collapsed to create a dichotomous variable. One item (#7), was not rated by frequency. There is no total score for the CPI but a CPI ratio, calculated the number of important activities engaged in often enough or too much (numerator) to the number of important activities (denominator), across participants as well as for each item. Scores range between 0 and 1 with higher scores indicating increased participation in activities that are meaningful to the individual (14). The CPI was validated through Rasch analysis in a sample of 1,163 individuals with a variety of diagnoses (13, 14), however results focused solely on a stroke population have not been previously reported for Part 1 of the CPI.

#### Stroke Impact Scale 3.0 Participation Subscale (SIS-P) and Visual Analog Stroke Recovery Scale

The SIS-P is the most frequently used scale to measure participation following stroke (15). The SIS-P is a self-report questionnaire containing 8 questions that ask the participant to rate how much of the time he or she has been limited in the past 4 weeks in work, social, productive activities and control over one's life (16). The responses to each question are scored on a scale of 1 (all of the time) to 5 (none of the time). Domain scores range from 0 to 100, with higher scores indicating that fewer problems (less impact) are perceived. Scores of <50 indicate limited participation (17, 18). The SIS also includes 1 item, presented in the form of a vertical visual analog scale (VAS), that assesses perceptions of overall stroke recovery, ranging from 0 = “no recovery” to 100 = “full recovery.” The SIS domains have high reliability, with Cronbach's alphas ranging from 0.83 to 0.90 and intraclass correlation coefficients (ICCs) ranging from 0.70 to 0.92 (19). Validity of the domains have been established through Rasch analysis (16). Concurrent validity (19) and construct validity have also been established (20). The SIS has been demonstrated to have good agreement between persons with stroke and proxies (21).

### Procedures

Participants were contacted by their preferred method (e-mail, phone, mail) 2 weeks before their 6-month post-discharge date to set up a time for a phone interview to complete the SIS-P, Stroke recovery scale and CPI. Questions were sent to the participant prior to the phone interview, so that they had them during the phone interview. All participants were living in the community. If the person was unable to participate in a phone interview, the interview was completed by proxy. Data were entered into a stroke research database using REDCap (Research Electronic Data Capture), a secure, web-based data management application (22). Participants with missing or incomplete assessment data were excluded from the analysis. The National Institutes Stroke Scale score (NIHSS) documented from the emergency department or upon admission to neurology, along with demographic, background, and stroke-related characteristics from rehabilitation admission or discharge including the Functional Independence Measure (FIM), were extracted from the electronic medical record into the stroke database and subsequently analyzed along with the 6-month measures.

### Statistical Analysis

Descriptive statistics including median, interquartile range, frequency, and percent, were used to summarize demographic variables, and the CPI and SIS-P questionnaire items. Visual inspection of histograms as well as the Shapiro-Wilk test were used to assess normality of continuous variables. Participant age was categorized into 66 years and older or 65 and younger. It should be noted that the age cut-off for defining young strokes is unclear. The World Health Organization (WHO) defines young stroke as under age 65, while other studies include age 65 and below (Sweden study). Since the official retirement age in the United States is 66, we chose to divide younger and older adults by those above, or at and below age 65.

Individual CPI and SIS-P items, the total SIS-P score, stroke recovery rating and CPI ratio score were all compared across the two age groups. The Wilcoxon rank-sum test was used to assess the association between age group and continuous variables while the chi-square or Fisher's Exact test, as appropriate, was used to assess the association between age group and discrete variables. CPI items that were significantly associated with age group at *p* < 0.05 were used as outcomes in multivariable logistic regression models to assess the independent effect of age on the participation item, controlling for ethnicity and discharge FIM total score. These items were collapsed into binary variables. The CPI items relating to doing an activity “enough” were collapsed into “enough or too much” vs. “not enough” while the item relating to frequency was collapsed into “with some frequency (1 to > 35 h)” vs. “none.” Co-variates and potential confounders were selected based on literature review and clinical knowledge. The correlation between the CPI ratio score and the total SIS score was assessed with Spearman's rank correlation coefficient. All *p*-values are two-sided with statistical significance evaluated at the 0.05 alpha level. All analyses were performed by a biostatistician in *R* Version 3.5.3 (Vienna, Austria) (23).

## Results

Of 273 inpatients who provided consent to be contacted on 6-month follow-up, ~36% (*n* = 99), responded and fully completed participation outcome measures. This resulted in a final sample that demonstrated relatively mild and some moderate neurological and cognitive/ language deficits. Table 1 summarizes demographic and clinical characteristics of the final sample.

**Table 1:** Demographic and clinical characteristics of the participants (*N* = 99).

**Characteristic**	**Entire sample, *n* = 99**	**Younger ≤ 65, *n* = 43**	**Older ≥ 66, *n* = 56**	***P***
**Age**	69 (55, 77.5)	53 (49.5, 61)	76 (71.8, 83.2)	<0.001^*^
**Sex** ***n*** **(%)**				1.000
Male	50 (50.5)	22 (51.2)	28 (50)	
Female	49 (49.5)	21 (48.8)	28 (50)	
**Ethnicity** ***n*** **(%)**				<0.001^*^
Caucasian	58 (58.6)	14 (32.6)	44 (78.6)	
Black	18 (18.2)	14 (32.6)	4 (7.1)	
Hispanic	10 (10.1)	9 (20.9)	1 (1.8)	
Asian/Pacific Islander	10 (10.1)	3 (7)	7 (12.5)	
Other	3 (3)	3 (7)	0 (0)	
**Education**				0.347
Less than high school	8 (8.1)	2 (4.65)	6 (10.7)	
Completed high school	26 (26.3)	14 (32.6)	12 (21.4)	
Some college	14 (14.1)	8 (18.6)	6 (10.7)	
College degree or higher	51 (51.5)	19 (44.2)	32 (57.1)	
**Length of Stay**	12 (8, 17)	13 (9, 19)	12 (8, 15.2)	0.214
**Side of Lesion** ***n*** **(%)**				0.199
Left hemisphere	42 (42.4)	18 (41.9)	24 (42.9)	
Right hemisphere	47 (47.5)	18 (41.9)	29 (51.8)	
Bilateral	10 (10.1)	7 (16.3)	3 (5.4)	
**Type of Stroke** ***n*** **(%)**				0.063
Ischemic	82 (82.8)	32 (74.4)	50 (89.2)	
Hemorrhagic	17 (17.1)	11 (25.6)	6 (10.7)	
**Prior TIA/CVA** ***n*** **(%)**	20 (20.2)	9 (20.9)	11 (19.6)	1.000
**Days post-CVA**	6 (4, 11)	7 (5, 13)	5 (3, 11)	0.037^*^
**NIHSS score**	4 (3, 8.5)	5 (3, 10.5)	4 (2, 6)	0.039^*^
**Discharge-home** ***n*** **(%)**	73 (74.5)	30 (71.4)	43 (76.8)	0.919
**Work prior to stroke** ***n*** **(%)**	36 (36.3)	25 (58)	11 (19.6)	<0.001^*^
**Work after stroke** ***n*** **(%)**	16 (16.2)	9 (20.9)	7 (12.5)	<0.001^*^
**Discharge FIM**				
Motor FIM	57 (49.5, 65.5)	59 (51.5, 65.5)	55.5 (47, 64)	−0.586
Cognitive FIM	31 (24, 34)	32 (24, 34)	31(24.8, 34)	−0.192
Total FIM score	88 (76.5, 99.5)	89 (80.5, 102)	87 (72.8, 97.2)	−0.787
**SIS total score**	65.6 (42.2, 90.6)	56.2 (31.2, 82.8)	70.3 (46.9, 90.6)	0.075
SIS <50 *n* (%)	33 (33.3)	18 (41.9)	15 (26.8)	0.12
**SIS stroke recovery**	75.0 (50.0, 85.0)	75.0 (50.0, 85.0)	72.5 (50.0, 86.2)	0.879
**CPI ratio score**	0.53 (0.22, 0.75)	0.45 (0.21, 0.69)	0.57 (0.24, 0.79)	0.132

* *P < 0.05*.

Compared with the final sample, those not included had greater language and cognitive disability as reflected by median [IQR] FIM discharge cognitive score (median = 28, [IQR = 21.0; 33.0] compared to 31 [IQR = 24.0; 34.0] and higher median [IQR] NIHSS scores 6 [3.00; 12.00] vs. 4 [3.00; 8.50]; *p* = 0.02. No significant differences were observed for the discharge motor FIM score, or other demographic variables such as age, sex, ethnicity, length of stay, or side of lesion.

Participants included in the final analysis (Table 1) had a median age of 69, equal representation of gender, 80% first time stroke, median NIHSS score of 4, a total discharge FIM score of 88 and were mostly Caucasian (59%). Twenty-seven percent of respondents were by proxy. Those with proxy respondents had significantly lower discharge FIM cognitive (median = 25 vs. 32), *p* < 0.001) and motor scores (50 vs. 59) *p* = 0.003.

The final sample was divided by age groups. The <65 age group demonstrated significantly more ethnic diversity than the older group, were admitted to inpatient rehabilitation after a longer number of days post-stroke and had a higher median NIHSS score. There were no significant differences between groups in sex, educational level, length of stay, side of lesion, proxy respondents, prior stroke, discharge home, or admission and discharge FIM scores.

### Comparison of Frequency of Participation Between Age Groups

#### CPI Frequency (Objective Participation)

Overall, both groups reported high frequency of participation with getting out and about, spending time with family, keeping in touch with family or friends, and engaging in hobbies or leisure activities. Least frequently engaged activities were participating in civic activities, self-help groups, clubs, and volunteer work. There were no significant differences between age groups in reported frequencies of activities with the exception of looking after children or providing care for a loved one, with older adults reporting less frequency compared to younger adults (*p* = 0.033). Table 2 summarizes the frequency of engagement in CPI activities (dichotomized into none vs. level of frequency combined).

**Table 2:** Community Participation Indicators (CPI): Descriptive Statistics of activities in younger and older adults post-stroke.

	**Younger < 65**				**Older >65**			
**Activity**	**Frequency % > none**	**Important %**	**% Doing activity enough**	**CPI Ratio**	**Frequency % > none**	**Important %**	**% Doing activity enough**	**CPI Ratio**
1. Get out and about	97.7	95.3	53.5	51	89.3	92.9	50.9	48
2. Spend time with family	86	90.7	76.7	74	85.7	90.9	74.5	72
3. Keep in touch with family by phone or internet	93	93	79.1	78	94.6	94.6	74.5	72
4. Spend time with friends	65.1	86	52.4	46	78.6	87.5	56.4	49
5. Keep in touch with friends by phone or internet	88.4	83.7	76.7	72	89.3	85.7	80.4	77
6. Go to parties, out to dinner, or other social activities	55.8	81.4	53.5	43	55.4	78.6	51.8	43
7. Spend time with a significant other or intimate partner	–	85.7	58.5	50	–	59.3	79.6	69
8. Work for money	23.3	86	37.2	27	16.1	35.7	71.4	20
9. Cook, clean, and look after your home	72.1	88.4	55.8	50	55.4	67.9	60.7	45
10. Manage household bills and expenses	67.4	85.7	71.4	67	67.9	80.4	75	71
11. Look after children or provide care for a loved one	30.2	62.8	58.1	33	14.3	32.1	80	39
12. Go to classes or participate in learning activities	20.9	65.1	51.2	25	28.6	45.5	73.2	40
13. Volunteer	11.6	55.8	53.5	21	14.3	37.5	76.8	38
14. Participate in religious or spiritual activities	55.8	74.4	51.2	38	40	48.2	66.1	30
15. Go to support groups or self-help meetings	7	41.9	65.1	17	3.6	25	76.8	7
16. Engage in hobbies or leisure activities	79.1	100	38.1	37	82.1	91.1	58.9	55
17. Go to movies, sporting events or entertainment events	48.8	86	44.2	35	48.2	60.7	67.9	47
18. Participate in sports or active recreation	76.7	90.7	39.5	36	73.2	82.1	55.4	46
19. Participate in community clubs or organizations	9.3	48.8	60.5	19	17.9	35.7	76.8	40
20. Participate in civic or political activities	7	20.9	79.1	0	8.9	28.6	78.6	25

*CPI ratio- Scores closer to 1.00 indicates greater participation in meaningful activities (both important and enough) (14)*.

#### Stroke Impact Scale (SIS-P) (Objective Participation and Perceived Stroke Recovery)

We observed no statistical evidence that older and younger subjects in this cohort differed between SIS-P individual item responses, total SIS-P scale or the SIS stroke recovery scale at 6 months. However, a large variation in both groups and a trend toward lower participation scores on the SIS-P for younger adults (*p* = 0.075) was observed. Overall, the scores on the SIS-P ranged from 6 to 100 in the younger group and 3 to 100 in the older group. A greater proportion of younger adults (42%) had a total SIS-P score below 50, while only 27% of older adults scored below 50. At the same time, ¼ of younger participants (26%) and 1/3 of older participants (36%) reported high levels of participation with scores >80. Similarly, there was a wide range of perceptions on the stroke recovery scale, ranging from 0 to 100. The distribution of scores however, was nearly equivalent across groups with 28–29% reporting a recovery of 80 or above. The relationship between perceived recovery and the SIS-P was stronger in the older group rho = 0.69, *p* = 0.001 than the younger group, rho = 0.45, *p* = 0.002). There was a moderate, positive correlation between the CPI ratio and SIS Total scores (rho = 0.54, *p* < 0.001).

### CPI Activity Importance and Engagement in Meaningful Activities (Subjective Participation)

Table 2 summarizes the proportion of participants in each age group who viewed an activity as important, and who reported an activity was being done enough. Overall, 13 items on the CPI were identified by at least 40% or more of the younger group as activities that were not done enough. In contrast, 5 items were identified by at least 40% of the older group as not being done enough. Table 2 also includes the average CPI ratio or the extent that items reflected engagement in meaningful activities (both important and enough) by each group.

Activities that were most important to people, generally included those that had high frequent engagement, however there were significant differences in some activities that were more important to younger adults. For example, compared to older adults, younger people more frequently identified important activities as spending time with a significant other or intimate partner *(p* = 0.009) working (*p* < 0.001), cooking (*p* < 0.031), looking after a loved one (*p* = 0.005), spiritual activities *(p* = 0.02), and entertainment (*p* = 0.01).

Participation in activities that are meaningful to the person in both groups as reflected by higher CPI ratio scores (important and doing enough) included spending time with family, keeping in touch with family or friends by phone or internet and managing household bills and expenses. In contrast, activities identified as important but not being done enough by both groups included a desire to get out and about more (46–49%), participate in social activities (46–48%) engage in hobbies and leisure (41–62%), participate in sports or recreation (45–60%). Younger adults were significantly more likely to indicate that they were not doing the following activities enough compared with older adults: Spend time with a significant other or intimate partner (*p* = 0.045), work for money *(p* = 0.001), look after children or provide care for a loved one (*p* = 0.033), go to classes or participate in learning activities *(p* = 0.040), volunteer, and go to movies, sporting events or entertainment events (*p* = 0.031). Although differences were observed at an individual item level, the average CPI ratio was not significantly different between age groups, although there was a slight trend toward younger participants reporting less participation in meaningful activities (median = 0.45) compared to older adults (median = 0.57).

### Association Between Participation Domains and Demographic and Clinical Variables

We did not find an association between acute stroke severity as measured by the NIHSS and participation restrictions (SIS-P or CPI). There was a weak positive association between the SIS-P and FIM total score (rho = 0.36, *p* = 0.0003) that was slightly stronger in the older group (0.43, *p* < 0.001) compared to the younger group (0.35, *p* < 0.05). The relationship between the CPI ratio score and FIM total score was also weak (rho = 0.20, *p* = 0.05). Upon closer analysis, this relationship only existed in the older group (rho = 0.43, *p* < 0.001) and not in the younger group.

#### CPI: Demographic Factors Influencing Desire for Participation

In multivariate logistic regression models, controlling for ethnicity, sex and FIM Total Score at 6 months following discharge, the independent effect of age group on desire for participation was maintained for the following items: “work for money” enough (OR = 3.83 (95% CI: 1.45, 10.09), *p* = 0.007) and “go to movies, sporting events or entertainment events” (OR = 3.03 (95% CI: 1.15, 7.97), *p* = 0.024). Subjects that were 66 or older had *higher odds* of saying they did the activity “work for money” and “Go to movies, sporting events or entertainment events” *enough* compared to those 65 and younger, controlling for ethnicity, gender and discharge FIM total score. We did not find an independent association of age for the other outcomes. This may have been due to the different distribution of ethnicity in the two age groups. Supplementary Figure 1 illustrates these differences graphically.

On the CPI frequency outcome of interest, subjects that were 66 or older had lower odds of saying they did the activity “look after children or provide care for a loved one” with some frequency compared to those 65 and younger, controlling for ethnicity, gender, and discharge FIM total (OR = 0.23 (95% CI: 0.07, 0.75), *p* = 0.015). See Supplementary Figure 2.

### Relationship Between SIS-P (Frequency) and CPI (Engagement in Meaningful Activities)

There was a moderate, positive correlation between the CPI ratio score (engagement in meaningful activities) and the SIS-P (frequency of participation) (rho = 0.54, *p* < 0.001). This relationship was slightly stronger in the older group (rho = 0.55, *p* = 0.000) as compared to the younger group (rho = 0.44, *p* < 0.001).

## Discussion

We compared both objective (frequency) and subjective dimensions (importance, doing activities enough) of participation in younger and older adults, 6 months after discharge from intensive inpatient stroke rehabilitation. Associations between participation, demographics, stroke severity and functional discharge status were explored and the relationship between objective and subjective participation measures (SIS-P and CPI) was examined. We discuss our findings for each these areas.

### Frequency of Participation Across Age Groups

Both age groups reported decreased frequency of participation particularly in social activities and entertainment events. There were however, no significant differences in perceived frequency of participation restrictions (SIS-P), activity participation (CPI), or perceived stroke recovery for those <65 compared with those >65. An exception was 1 item on the CPI; look after children or caring for a loved one, that was less frequent for older adults. The similarities observed between age groups in frequency of participation might be confounded by the effects of aging in older adults. Increases in perceived participation restrictions have been reported for healthy adults after the age of 80 years old (24). Studies have found that older adults do not score at the maximum level on participation instruments (25, 26). For example, Lai et al. (26) found that healthy older adults had an average score of 86/100 on the SIS-P. Similar to Lai et al. (26) we found that the average SIS-P for people with stroke was lower than that reported for healthy adults. This however, raises questions about whether the lower frequency for some items on the CPI or SIS-P may be partially related to normal aging rather than to the effects of stroke. If some older adults have a lower participation baseline prior to the stroke, comparison to younger adults after stroke may not necessarily reflect true differences.

Younger adults demonstrated a trend toward reporting more participation restrictions than the older group (42 vs. 27%) on the SIS-P, despite similar perceived stroke recovery ratings. This suggests that the younger group may have had higher expectations for performance or engagement in life activities. Therefore, greater participation restrictions were perceived despite relatively good stroke recovery.

The lack of an association in perceived frequency of participation and age in this sample is in contrast to other studies that have found that older age is associated with more participation restrictions compared to those who are younger (27, 28). For example, researchers in the Netherlands found that stroke survivors age 70 and above reported greater participation restrictions after 1 year than those below age 70. It is difficult to compare studies due to differences in participation outcome measures, time points and variations in age groups, however, differences in our findings may be at least in part to variations in sample characteristics. Our sample only included those who participated in intensive short-term rehabilitation. The cognitive and motor functional level of both age groups was similar on rehabilitation admission and discharge, whereas in other studies, there were significant differences in cognitive and functional skills between younger and older age groups shortly following stroke (9).

### Subjective Aspect of Participation

Despite similarities between age groups for frequency of participation, important differences were observed in the value and desire for activity engagement across selected items on the CPI. Given the increased incidence of young stroke discussed earlier, this finding implies that stroke rehabilitation may need to tailor programs to meet the different priorities of younger and older adults. For example, 43% of younger adults identified *caring for a child or loved one* as an activity that is not done enough, 6 months post-stroke. Childcare is particularly relevant to participation immediately after discharge from inpatient rehabilitation, however there is very little attention or research in this area after a stroke (29). Greater attention to the priorities of younger people with stroke may be needed earlier in the rehabilitation process.

The differences observed between age groups also implies that some modifications in item content of participation measures for younger stroke survivors should be considered. Items that were significantly more meaningful for younger adults could be expanded. For example, a single item related to employment or childcare is likely insufficient in measuring the impact of stroke on participation in younger adults. Follow-up qualitative interviews related to these areas could provide further insights into the experiences and perspectives of young stroke survivors that could further shape development of participation assessment tools and intervention programs.

The differences in the subjective aspects of participation observed across several CPI activities highlight the need to assess both dimensions of participation, particularly at an individual activity level. This is consistent with the observations and findings of others (11).

Subjective appraisal of activity importance and desire for change (not doing an activity enough) provides key insights into priorities and valued activities for the individual that can help interpret the significance of reported activity frequency. Questions that ask if activities are carried out often enough is a unique aspect of the CPI and as Plow et al. (14) observed, is different than asking about activity satisfaction. For example, a person may be dissatisfied with their level of participation in household activities but at the same time, may not be interested in increasing engagement because other activities are more important to them. Measures of satisfaction have been used to assess the subjective aspect of participation, however inclusion of importance and desire for doing an activity more often, provides additional information on the subjective dimension of participation that may differ across ages and individuals. Since engagement in personally meaningful activities is associated with improvement in emotional well-being post-stroke and quality of life (30), information on valued and desired activities is essential for client centered treatment planning and goal setting in rehabilitation.

### Association Between Participation, Demographic, and Clinical Variables

The variations we observed in participation 6 months post-stroke was not associated with acute stroke severity. This is similar to that reported by other studies with mild-moderate stroke populations and age groups below an average of 65 years (17, 31). Additionally, frequency of participation restrictions (SIS-P) was weakly associated with functional discharge level (FIM). No relationship, however, was observed between participation in meaningful activities (CPI ratio) and functional discharge level (FIM) for younger adults. This is likely because perceptions of participation restrictions may be influenced more by the context of a person's life including community and home environment, individual preferences, lifestyle and expectations rather than level of impairment or ability to perform specific activities (12). This is particularly true for younger adults. Although a person may have a mild stroke or has achieved a high functional level following intensive rehabilitation, our results suggest that participation should still be monitored.

We further demonstrated that the desire to participate in some activities such as work or entertainment events was independently associated with age and was not explained by functional dependency level, sex, or ethnicity whereas desire for participation in other activities may have been possibly confounded by the relationship between ethnicity and age group. Different factors may therefore contribute to participation in different types of activities. Our younger group had greater ethnic diversity, mirroring the demographic profile of people with younger adults reported in the literature (4). The significant differences in minority representation in the younger group suggests that there might be other factors such as cultural preferences influencing participation. It also raises issues of possible health disparities that could restrict participation. Future studies examining differences in participation among age groups should further investigate ethnicity or cultural differences that may influence activity importance and desire for participation in select activities.

### Relationship Between SIS-P (Frequency) and CPI (Engagement in Meaningful Activities)

The relationship between frequency of participation restrictions (SIS-P) and subjective participation in valued and meaningful activities (CPI ratio) was moderate. This is consistent with other studies that have reported a moderate association between the subjective and objective aspects of participation (11). The correlation between the CPI ratio score to the established SIS-P score also supports the concurrent validity of the CPI as a participation measure. Although ~29% of both participation scales overlap, this finding indicates that each scale taps information that is unique or not captured by the other. This further supports the need to use multiple measures to provide a comprehensive assessment of different aspects of participation. This is consistent with recommendations by others (11, 32).

## Limitations

We acknowledge several limitations. Our sample consisted primarily of people with a mild-moderate stroke from a single inpatient rehabilitation unit within an academic medical center. A wider range of people with stroke and a larger sample size across different locations or facilities would allow for greater generalizability of results.

The small percentage of people (36%) who completed all follow-up measures is also a limitation. It was observed that the included sample was similar to the excluded sample, with the exception of cognitive/language deficits, however, this attrition indicates that results cannot be generalized to those with more significant cognitive or language deficits. Similarly, when necessary, a proxy completed follow up phone interviews. Those who had a proxy complete the interview also had lower language/cognitive abilities, and this could have led to bias in responses. Research on the SIS has found that observed biases between the individual and proxy were small and not clinically meaningful (21), however proxy agreement for the CPI has not been investigated for people with stroke.

Finally, it should also be noted that although both groups had a median NIHSS score in the mild range, the younger group had significantly greater stroke severity than the older group, more ethnic diversity and a higher percentage of hemorrhagic stroke (although not significantly different from the older group). Although we found no association between stroke severity and participation, these differences should be noted. Investigation of the effect of different types of stroke or specific cognitive and motor impairments on participation might provide further insights into factors impacting participation.

It should also be noted that while the CPI-part 1, was used to assess the subjective aspect of participation, it focused on importance and desire to increase engagement in participation. Other areas of subjective participation such as perceived autonomy (CPI-part 2) were not included in this study. While the CPI is quick and feasible to use, it is a survey instrument. Qualitative interviews could provide greater insights and a deeper understanding of the client's subjective perspective and experiences regarding participation after a stroke. Although cognitive interviewing has been reported with the CPI, to clarify the wording of questions and response format (33), interviews of people with stroke regarding their thoughts about the content of CPI items could further validate it as a tool.

## Conclusions

This is the first study to describe use of the CPI in people post-stroke 6 months following intensive rehabilitation. It highlights the unique aspects of the CPI and the merit of combining both frequency ratings and subjective appraisal of activity engagement for a deeper understanding of perceived participation restrictions. The value of examining the subjective dimension of participation at an individual activity level was demonstrated by comparing activity importance and desire for increased participation in those <65 and > 65. Despite similar perceived impact of stroke, stroke recovery and frequency rating, differences in age groups were most apparent for the subjective dimension of participation in selected items or activities. These differences suggest that stroke rehabilitation may need to tailor assessment and treatment programs to meet the different priorities of younger and older adults. Total frequency scores tended to mask individual differences in activity engagement. This suggests that clinicians should therefore focus on identifying individual activities or combinations of activities that are valued and that the person is motivated to change.

The desire to engage in some types of activities was independently associated with age, while other activities were not associated with age. Since there tends to be a greater minority representation in people with younger stroke, the influence of ethnicity and culture on participation needs further exploration. Additional research examining differences in participation among people in different age groups, including healthy older adults is needed. In addition to age, other groups differences such as those with low and high motor or cognitive impairments could be compared. Pre-stroke employment status could also be examined to explore how perceived participation restrictions after a stroke are related to pre-stroke participation.

Short term intensive rehabilitation focuses on discharge planning and increased independence in activities of daily living. Broader participation goals including the ability to fulfill family and life roles, integrate into the community, and engage in meaningful life activities require continued support. Participation outcomes may be optimized by monitoring and assessing participation post-stroke with follow up questionnaires after 6 months, such as the CPI. The CPI provides a strong foundation for implementation of individually tailored, client centered rehabilitation interventions, aimed at increasing engagement in meaningful activities when participation needs are identified.

## Data Availability Statement

The datasets used for this manuscript are not publicly available because it is still part of an active IRB research protocol. Requests to access the datasets should be directed to the corresponding author.

## Ethics Statement

This study was carried out in accordance with the ethical standards and approval of the Institutional Review Board of the participating institution with written informed consent from all subjects. All subjects gave written informed consent in accordance with the Declaration of Helsinki.

## Author Contributions

JT was involved the study concept and design, interpretation, manuscript development, and writing. GA and LG were involved in data analysis and interpretation and writing. AJ and MO'D were involved in conceptualization of results and manuscript editing. All authors reviewed final draft for intellectual content and revisions.

### Conflict of Interest

The authors declare that the research was conducted in the absence of any commercial or financial relationships that could be construed as a potential conflict of interest.

## Abbreviations

CPI: Community Participation Indicators
NIHSS: National Institute of Health Stroke Scale
SIS-P, Stroke Impact Scale: Participation domain
IRU: Inpatient rehabilitation unit
FIM: Functional Independence Measure.
