# Supplementary figures and images for: Participation in Younger and Older Adults Post-stroke: Frequency, Importance, and Desirability of Engagement in Activities
Source: Front Neurol. 2019 Oct 18;10:1108. doi: 10.3389/fneur.2019.01108 (PMC6813672; doi:10.3389/fneur.2019.01108)

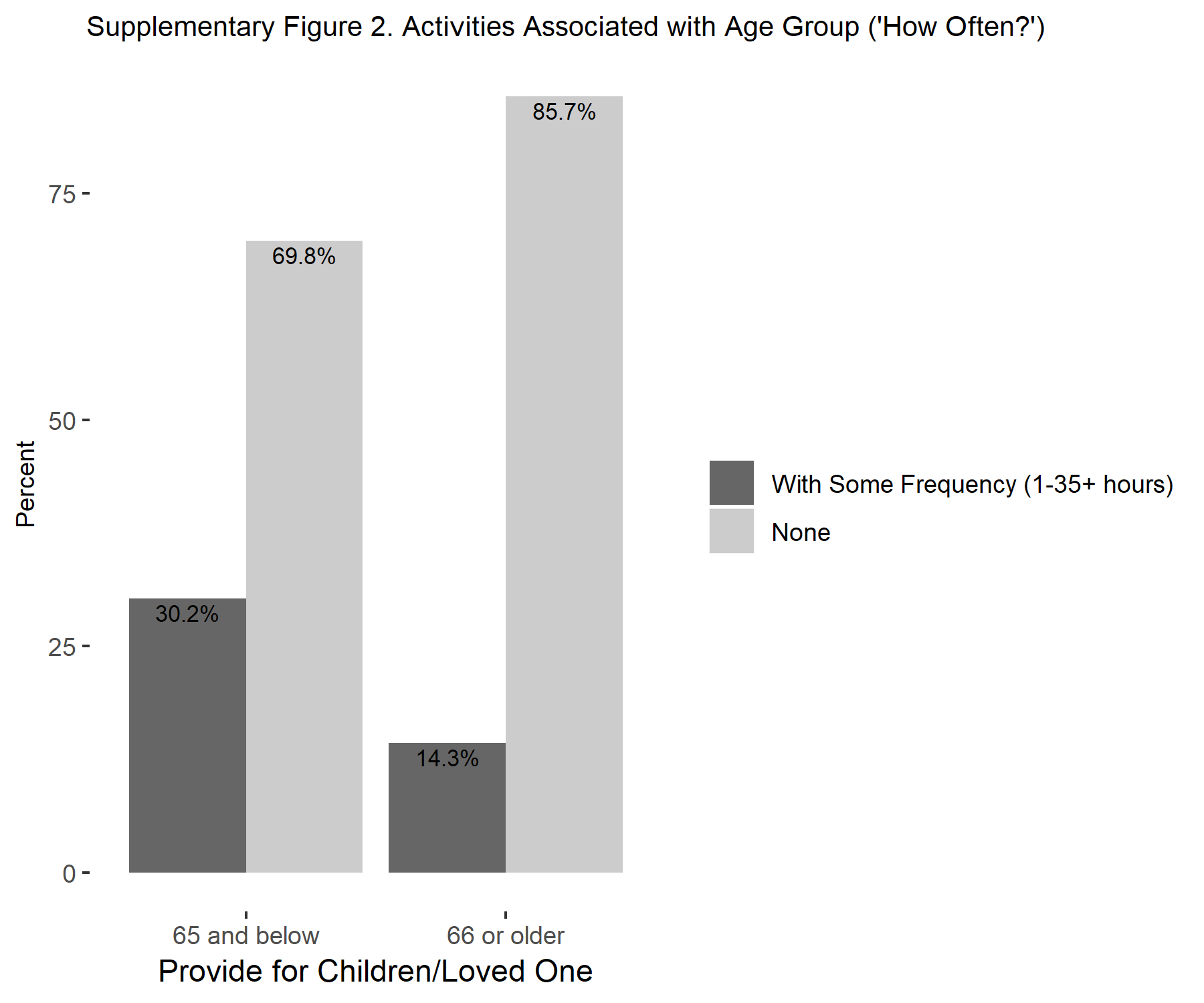

Supplement: Supplementary file 1 [file Data_Sheet_1.ZIP › freq_plot.tiff]

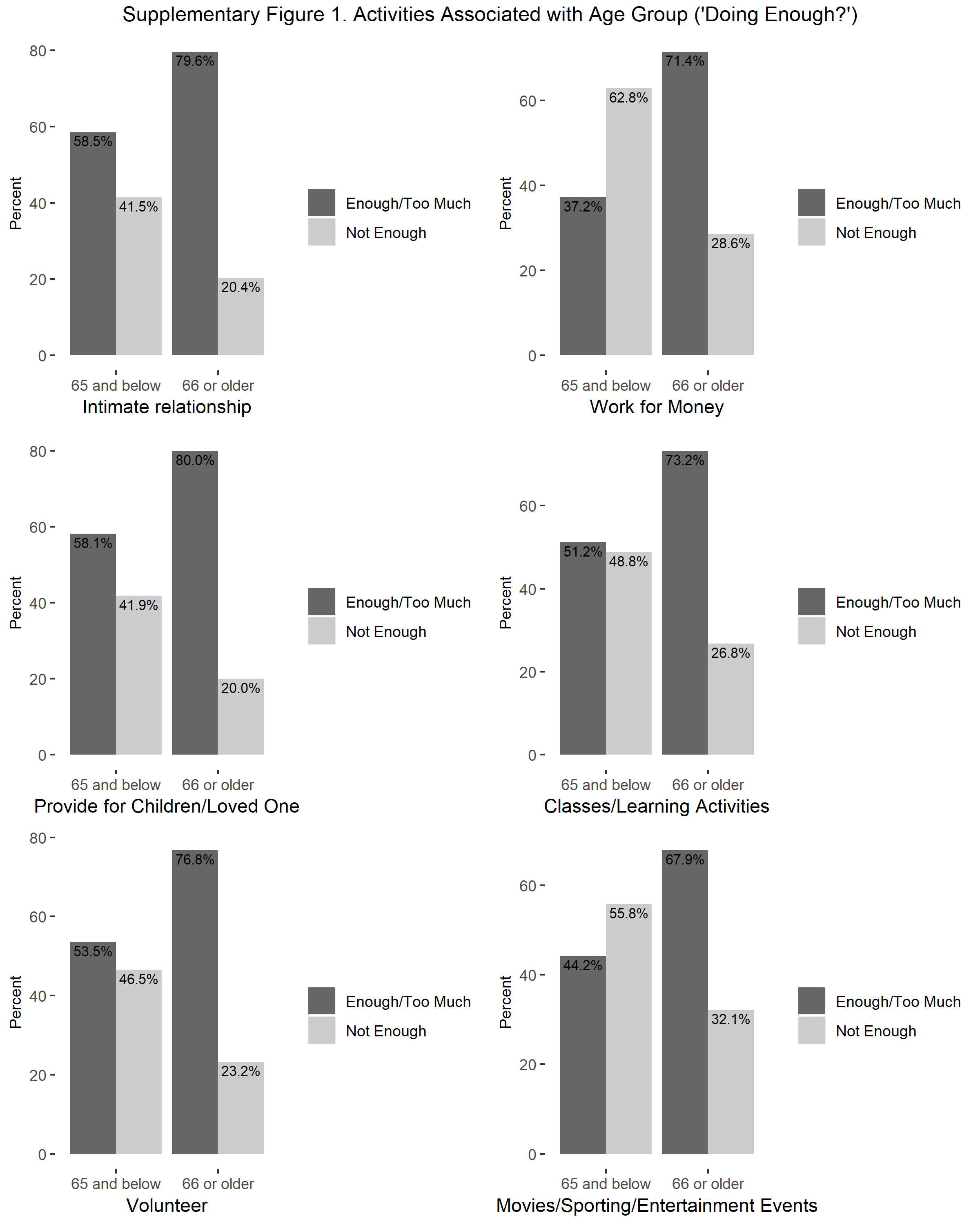

Supplement: Supplementary file 1 [file Data_Sheet_1.ZIP › panel_fin.tiff]
